# Glaucoma and risk factors three years after congenital cataract surgery

**DOI:** 10.1186/s12886-022-02343-9

**Published:** 2022-03-12

**Authors:** Zuhui Zhang, Yana Fu, Jiajun Wang, Xinpei Ji, Zhangliang Li, Yinying Zhao, Pingjun Chang, Yun-e Zhao

**Affiliations:** 1 Eye Hospital and School of Ophthalmology and Optometry, Wenzhou Medical University, Wenzhou, Zhejiang China; 2 National Clinical Research Center for Ocular Disease, Wenzhou, Zhejiang China; 3 Eye Hospital of Wenzhou Medical University, 618 East Fengqi Road, Hangzhou, 310000 Zhejiang China

**Keywords:** Congenital cataract surgery, Glaucoma-related adverse events, Incidence, Risk factors

## Abstract

**Background:**

This study aimed to identify the incidence of and risk factors for postoperative glaucoma-related adverse events at various time points after congenital cataract surgery.

**Methods:**

This retrospective cohort study enrolled 259 eyes from 174 patients (surgical age ≤ 7 years) who underwent congenital cataract surgery. All surgical procedures were conducted at the Eye Hospital of Wenzhou Medical University between May 2011 and March 2019. Patients were classified into group 1 [primary intraocular lens (IOL) implantation, *N* = 111 eyes], group 2 (secondary IOL implantation, *N* = 85 eyes), and group 3 (no IOL implantation, *N* = 63 eyes). We recorded demographic factors and incidence and risk factors for glaucoma-related adverse events.

**Results:**

Glaucoma-related adverse events occurred in 21 (8.1%) eyes, whereas 27 (10.4%) eyes developed steroid-induced ocular hypertension. The percentage of glaucoma-related adverse events was 0%, 1.2%, 1.2%, 1.6%, 4.0%, and 8.9% at 1 month, 6 months, 1 year, 2 years, 3 years and 4 years after surgery, respectively. Sixteen (18.8%), five (7.9%), and zero eyes developed glaucoma-related adverse events in groups 2, 3, and 1, respectively. Family history of congenital cataract [hazard ratio (HR), 50.463; 95% confidence interval (CI), 7.051–361.139; *P* < 0.001], preoperative central corneal thickness (CCT) [HR, 1.021; 95% CI, 1.009–1.034; *P* = 0.001], preoperative horizontal corneal diameter (HCD) [HR, 3.922; 95% CI, 1.558–9.804; *P* = 0.004], and preoperative lens thickness (LT) [HR, 3.745; 95% CI, 1.344–10.417; *P* = 0.012] were identified as predictors of postoperative glaucoma-related adverse events.

**Conclusions:**

Family history of congenital cataract, thicker preoperative CCT, smaller preoperative HCD, and thinner preoperative LT are the main risk factors of postoperative glaucoma-related adverse events. Regular monitoring of children after cataract surgery with these risk factors may help ophthalmologists detect susceptible individuals and provide timely interventions in the clinic.

## Background

Childhood glaucoma that develops after cataract surgery is classified as glaucoma following congenital cataract surgery [1]. Postoperative childhood glaucoma is a common and severe complication that can lead to blindness, despite immense progress in surgical techniques [2]. Glaucoma occurs in 6.0%–58.7% of children after congenital cataract surgery, depending on the age, follow-up time, and definition employed by various studies [3–11]. Elevation in intraocular pressure (IOP) is an early sign of postoperative glaucoma [12–14]. Glaucoma does not always follow a slow course, as it can develop quickly. However, if affected children are not followed up regularly, the diagnosis may be delayed.

As the ocular examination is often complicated in children, IOP and optic disc examinations are usually performed, whereas the visual field cannot be determined, further complicating the diagnosis of postoperative glaucoma. Therefore, the Infant Aphakia Treatment Study (IATS) classified both glaucoma and glaucoma suspect after congenital cataract surgery as glaucoma-related adverse events [15].

Studies have shown that intraocular lens (IOL) implantation, young age, cataract type, corneal diameter, central corneal thickness (CCT), and persistent foetal vasculature (PFV) may be associated with childhood glaucoma after surgery [4–8, 11, 16–19]. However, these risk factors are still controversial, and the most critical risk factors for glaucoma after cataract surgery are still unknown.

Children who undergo cataract surgery are routinely treated with topical corticosteroids, which renders them susceptible to steroid-induced ocular hypertension [12]. However, the correlation between steroid-induced ocular hypertension and late-onset open-angle glaucoma remains unclear.

In this study, we grouped patients according to the presence or absence of IOL implantation and compared outcomes among patients who had undergone primary, secondary, and no IOL implantation. The purpose of this study was to identify the incidence of and main risk factors for glaucoma-related adverse events at various time points after congenital cataract surgery.

## Methods

### Patient selection

This study enrolled children with congenital cataracts who underwent lensectomy combined with limited anterior vitrectomy, with or without IOL implantation at the Eye Hospital of Wenzhou Medical University (Hangzhou Branch) from May 2011 to March 2019. The inclusion criteria were (1) congenital or developmental cataract, (2) age ≤ 7 years, (3) need for surgical treatments with lens opacity obscuring the red reflex with dilated pupils, (4) all surgical procedures were performed by an experienced ophthalmologist (Yun-e Zhao), and (5) followed up for ≥ 12 months. The exclusion criteria were (1) preoperative IOP > 21 mmHg; (2) eyes concurrent with glaucoma, uveitis, retinopathy of prematurity, anterior segment dysgenesis, and systemic disorders (such as Marfan’s syndrome); and (3) history of ocular trauma and previous ocular surgery.

The choice of surgical methods generally followed such principles. Cataract removal was performed within 6 months of age for both unilateral and bilateral cataract children, and within 6 months to 1.5 years old for bilateral cataract children, then second-stage IOL implantation at about 2 years of age. Primary IOL implantation was performed for children over 6 months old with unilateral cataract and over 2 years old with bilateral cataract.

Patients were classified into three groups. Group 1 included eyes of primary IOL implantation. Group 2 included eyes of aphakic at the first stage and secondary IOL implantation. Group 3 included aphakic eyes.

This study adhered to the tenets of the Declaration of Helsinki and was approved by the Ethical Review Committee of Wenzhou Medical University (approval number: 2020–042-k-37–01). This study is registered on www.clinicaltrial.gov (NCT04521907).

### Ocular parameters

The CCT, axial length (AL), anterior chamber depth (ACD), lens thickness (LT), and horizontal corneal diameter (HCD) were measured, and the patients’ demographic data were recorded. CCT was measured via a handheld ultrasonic pachymeter (PachPen, Accutome, Inc., PA, USA). The AL, ACD, and LT were measured using an A-scan (Axis Nano, Quantel Medical, Cournon, France) under sedation before surgery. HCD was measured at the beginning of surgery using callipers. Children with incomplete lens opacity underwent fundus examinations, which then were completed via a Retcam (Retcam3, Clarity, USA) under sedation before surgery. Retcam was carried out in all lens opacity children 1 month after surgery. IOP was obtained using a handheld tonometer (Icare Finland Oy, Finland). Furthermore, the CCT was measured 3 times. AL, ACD, and LT were measured 10 times, and IOP was measured 6 times for each patient; the mean values were record.

### Surgical technique

All surgeries were performed by the same experienced congenital cataract surgeon (Yun-e Zhao) under general anaesthesia using the 23G vitrectomy mode of Accurus or Centurion vision system (Alcon Laboratories, Inc., TX, USA) with a cut-rate of 2,000 per min and vacuum of 350 mmHg. For children younger than 3 years old, the anterior vitrectorhexis about 5.0 mm was performed through 1.0 mm precise corneal incision at 10 and 2 o 'clock; the cortex was aspirated by the vitrector, then the posterior vitrectorhexis of about 3.5 mm, and followed by limited anterior vitreous. Then, left aphakic or implanted IOL into the capsule bag. Different from the above, for children over 3 years old, the anterior capsulorhexis and the posterior capsulorhexis or vitrectorhexis were performed. For second-stage IOL implantation, the capsular bag was reopened followed by in-the bag IOL implantation. The IOL was fixed in the ciliary sulcus if the capsule was insufficient to implant the IOL into the capsular bag.

### Definitions

The definitions for glaucoma, glaucoma suspect, and glaucoma-related adverse events provided by the IATS [19] and the Childhood Glaucoma Research Network (CGRN) [1] were applied rigorously. A study eye was diagnosed as glaucoma if the IOP was > 21 mmHg with ≥ 1 of the following anatomical changes: (1) corneal enlargement, (2) asymmetrical progressive myopic shift coupled with enlargement of the corneal diameter and/or axial length, (3) increased optic nerve cupping defined as an increase of ≥ 0.2 in the cup-to-disc ratio, or (4) a surgical procedure was performed for IOP control. A study eye was diagnosed as a glaucoma suspect if there was either: (1) recording of two consecutive IOP measurements > 21 mmHg on different dates after topical corticosteroids had been discontinued without any of the anatomical changes listed above for glaucoma or (2) glaucoma medication was used to control IOP without any of the anatomical changes listed above. Glaucoma-related adverse events were defined as glaucoma plus glaucoma suspect. Steroid-induced ocular hypertension was defined as a recording of IOP measurements > 21 mmHg at least once after topical corticosteroids had been continued without any of the anatomical changes listed above for glaucoma and returned to normal after topical corticosteroids had been discontinued.

### Statistical analysis

Statistical analyses were performed using SPSS version 23.0 (IBM Corp., Armonk, NY, USA). The normality of data distributions was analysed using the Kolmogorov–Smirnov test, and the abnormality of data distributions was analysed using non-parametric statistical analyses. Values are expressed as the mean ± standard deviation (SD) or range or median (interquartile range [IQR]). The independent samples t-test or the Mann–Whitney U-test was employed to compare the parameters among the different groups. The chi-square test or Fisher’s exact test was used to compare the categorical variables. The generalised estimating equation was used to adjust the difference of unilateral and bilateral cataract. The demographic, ocular, and systemic predictors of incident glaucoma were examined using Cox proportional hazards regression to generate hazard ratios (HRs) and associated 95% confidence intervals (CIs). A *p*-value < 0.05 was considered statistically significant.

## Results

### Characteristic features and glaucoma-related adverse events after primary IOL implantation, secondary IOL implantation, and without IOL implantation

Data from 259 eyes (174 patients, 96 men) were analysed. The median follow-up period was 43.0 (IQR, 31.0–55.0) months. The median age at primary surgery was 6.0 (IQR, 3.0–26.0) months.

Group 1 included 111 eyes that underwent cataract surgery with primary IOL implantation (109 eyes in the bag and 2 in the sulcus), group 2 comprised 85 eyes that underwent primary cataract removal and secondary IOL implantation (68 in the bag and 17 in the sulcus), and group 3 included 63 eyes that underwent cataract removal without IOL implantation. The baseline demographics of the three groups are shown in Table 1. Because the unilateral cataract difference among the three groups is significant (all *P* < 0.05), a generalised estimating equation was used to adjust the correlation between the eyes.

**Table 1:** Clinical characteristics of patients with different surgical procedures

	**Group 1**	**Group 2**	**Group 3**	***P-value 1***	***P-value 2***	***P-value 3***	* P-value 1	* P-value 2	* P-value 3
Eyes, n	111	85	63						
Right eye, n	57	43	34						
Unilateral cataract, n (%)	61 (55.0)	22 (25.9)	7(11.1)	< 0.001	< 0.001	0.025			
IOL implantation, n									
In-the-bag	109	68	–						
Sulcus	2	17	–						
History of prematurity, n (%)	7 (6.3)	13 (15.3)	4 (6.4)	0.039	1.000	0.092	0.287	0.518	0.175
FHCC, n (%)	7 (6.3)	12 (14.1)	8 (12.7)	0.067	0.149	0.803	0.525	0.838	0.621
PFV, n (%)	16 (14.4)	17(20.0)	10(15.9)	0.300	0.795	0.520	0.015	0.037	0.566
Age at surgery (months), Median (IQR)									
Primary surgery	34.0 (15.0–46.0)	4.0 (3.0–5.0)	3.0 (2.0–4.0)	< 0.001	< 0.001	0.008	< 0.001	< 0.001	0.045
Secondary surgery	–	34.0 (24.0–37.0)	–						
Follow-up (months), Median (IQR)	39.0 (29.0–53.0)	50.0 (44.0–62.0)	35.0 (27.0–44.0)	< 0.001	0.050	< 0.001	< 0.001	0.030	< 0.001
IOP (mmHg), Mean ± SD									
Baseline 1	12.9 ± 3.1	12.4 ± 2.6	10.8 ± 2.7	0.176	< 0.001	< 0.001	0.292	< 0.001	0.002
Baseline 2	–	13.9 ± 2.7	–						
Latest follow-up	14.6 ± 3.1	16.8 ± 4.0	14.4 ± 3.1	< 0.001	0.635	< 0.001	0.001	0.503	< 0.001
Pre-op HCD (mm), Mean ± SD	10.4 ± 0.6	9.4 ± 0.7	9.4 ± 1.0	< 0.001	< 0.001	0.982	< 0.001	< 0.001	0.903
Pre-op CCT (μm), Mean ± SD	541.1 ± 44.5	564.8 ± 49.7	574.6 ± 46.9	0.001	< 0.001	0.230	0.012	0.002	0.359
Pre-op ACD (mm), Mean ± SD	2.9 ± 0.6	2.3 ± 0.4	2.4 ± 0.5	< 0.001	< 0.001	0.782	< 0.001	< 0.001	0.874
Pre-op LT (mm), Mean ± SD	3.7 ± 0.9	3.4 ± 1.0	3.1 ± 1.1	0.185	0.003	0.095	0.337	0.070	0.218
Pre-op AL (mm), Mean ± SD	21.5 ± 2.0	18.7 ± 1.1	17.9 ± 1.5	< 0.001	< 0.001	< 0.001	< 0.001	< 0.001	0.006
Glaucoma, n (%)	0 (0)	6 (7.1)	4 (6.3)	0.006	0.016	1.000	0.025	0.144	0.894
Glaucoma suspect, n (%)	0 (0)	10 (11.8)	1 (1.6)	< 0.001	0.362	0.025	0.010	0.304	0.058
Glaucoma-related adverse events, n (%)	0 (0)	16 (18.8)	5 (7.9)	< 0.001	0.006	0.061	< 0.001	0.070	0.127
Steroid-induced ocular hypertension, n (%)	5 (4.5)	17 (20.0)	5 (7.9)	0.001	0.499	0.041	0.009	0.211	0.038

*P*-value 1: Group 1 vs Group 2; P-value 2: Group 1 vs Group 3; P-value 3: Group 2 vs Group 3

^*^ *P* values adjusted for unilateral cataract difference by generalised estimating equation

Baseline 1, primary preoperative IOP; Baseline 2, secondary preoperative IOP

*IOL* Intraocular lens, *FHCC* Family history of congenital cataract, *PFV* Persistent fetal vasculature, *IQR* Interquartile range, *IOP* Intraocular pressure, *Pre-op* Preoperative, *HCD* Horizontal corneal diameter, *CCT* Central corneal thickness, *ACD* Anterior chamber depth, *LT* Lens thickness, *AL* Axial length

Five eyes in group 2 had open-angle glaucoma, and one eye had angle-closure glaucoma, which occurred before secondary IOL implantation. Two eyes (3.2%) in group 3 developed angle-closure glaucoma, and two eyes (3.2%) developed open-angle glaucoma. Sixteen eyes (18.8%) in group 2 developed glaucoma-related adverse events, five eyes (7.9%) in group 3 developed glaucoma-related adverse events, but no eyes were affected in group 1. Compared with the other groups, glaucoma-related adverse events rarely occurred in group 1 (i.e., after primary IOL implantation). The baseline average IOP before surgery and the last obtained IOP value in the three groups were within the normal range. However, the last obtained IOP value in group 2 was higher than group 1 (*P* = 0.001) and group 3 (*P* < 0.001).

Five eyes (4.5%) in group 1, 17 eyes (20.0%) in group 2, and 5 eyes (7.9%) in group 3 developed steroid-induced ocular hypertension. The difference between group 2 and the other two groups was statistically significant (all *P* < 0.05).

The median age at primary surgery was 34.0 (IQR, 15.0–46.0) months in group 1, 4.0 (IQR, 3.0–5.0) months in group 2, and 3.0 (IQR, 2.0–4.0) months in group 3. The differences among three groups were statistically significant (all *P* < 0.05). The median age at secondary surgery in group 2 was 34.0 (IQR, 24.0–37.0) months. The median follow-up periods were 39.0 (IQR, 29.0–53.0) months in group 1, 50.0 (IQR, 44.0–62.0) months in group 2, and 35.0 (IQR, 27.0–44.0) months in group 3. The differences in the follow-up time among three groups were statistically significant (all *P* < 0.05).

Compared with the other two groups, group 1 included patients with less PFV percentage, larger preoperative HCD, thinner preoperative CCT, deeper preoperative ACD, and longer preoperative AL (all *P* < 0.05). History of prematurity, family history of congenital cataract, and preoperative LT were not significantly different among three groups (all *P* > 0.05).

### Incidence of glaucoma-related adverse events and steroid-induced ocular hypertension

**Fig. 1:**
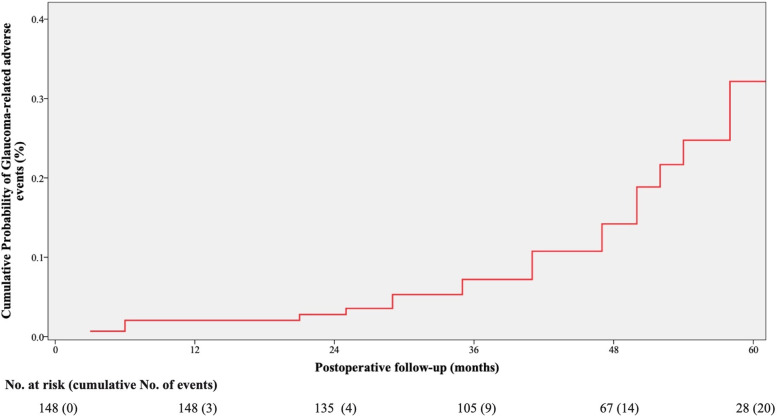
Cox proportional hazards regression analysis illustrating the cumulative probability of glaucoma-related adverse events

In this study, overall 10 (3.9%) eyes developed glaucoma, 11 (4.2%) eyes developed glaucoma suspect, so 21 (8.1%) eyes experienced glaucoma-related adverse events. Table 2 presented the percentage of postoperative glaucoma, glaucoma suspect, and glaucoma-related adverse events at various time points. The percentage of postoperative glaucoma was 0%, 1.2%, 1.2%, 1.2%, 2.7%, and 3.8% at 1 month, 6 months, 1 year, 2 years, 3 years, and 4 years, respectively. Similarly, the percentage of postoperative glaucoma suspect ranged from 0% to 5.1% at various time points. The percentage of postoperative glaucoma-related adverse events was 0%, 1.2%, 1.2%, 1.6%, 4.0%, and 8.9% at 1 month, 6 months, 1 year, 2 years, 3 years, and 4 years, respectively. Figure 1 showed the cumulative probability of glaucoma-related adverse events after cataract surgery.

**Table 2:** Percentage of glaucoma, glaucoma suspect, and glaucoma-related adverse events

	**1 month**	**6 months**	**1 year**	**2 years**	**3 years**	**4 years**
**Glaucoma, % (n)**	0 (0)	1.2 (3)	1.2 (3)	1.2 (3)	2.7 (6)	3.8 (6)
**Glaucoma Suspect, % (n)**	0 (0)	0 (0)	0 (0)	0.4 (1)	1.3 (3)	5.1 (8)
**Glaucoma-related adverse events, % (n)**	0 (0)	1.2 (3)	1.2 (3)	1.6 (4)	4.0 (9)	8.9 (14)

1 month, 6 months, and 1 year: number of eyes, *N* = 259; 2 years: number of eyes, *N* = 257; 3 years: number of eyes, *N* = 225; 4 years: number of eyes, *N* = 158

In Table 3, three eyes were diagnosed with angle-closure glaucoma within 6 months of primary surgery, and seven eyes developed open-angle glaucoma 54.0(IQR, 29.0–58.0) months after primary surgery. The mean time to diagnosis was 41.8 ± 9.1 months for the glaucoma suspect. Overall, open-angle glaucoma (7 eyes) and glaucoma suspect (11 eyes) were controlled with topical antiglaucoma medications, while angle-closure glaucoma (3 eyes) needed anti-glaucoma surgery.

**Table 3:** Clinical findings of glaucoma-related adverse events eyes after congenital cataract surgery patient

﻿Patient	Group	Sex	Eye	Glaucoma type	Age at primary surgery (months)	﻿ Age at secondary surgery (months)	IOL implantation	Age at glaucoma diagnosis (months)	﻿IOP max (mmHg)	﻿Anti-glaucoma
**1**	2	Female	Right	OAG	7	35	Sulcus	29	30	Topical antiglaucoma medications
**1**	2	Female	Left	OAG	7	35	Sulcus	29	31	Topical antiglaucoma medications
**2**	3	Male	Right	OAG	2	-	-	58	30	Topical antiglaucoma medications
**2**	3	Male	Left	OAG	2	-	-	58	27	Topical antiglaucoma medications
**3**	2	Male	Left	OAG	1	17	Sulcus	62	28	Topical antiglaucoma medications
**4**	2	Male	Right	OAG	5	39	Sulcus	54	35	Topical antiglaucoma medications
**5**	2	Female	Left	OAG	8	22	Sulcus	25	28	Topical antiglaucoma medications
**6**	2	Female	Right	ACG	2	27	Sulcus	6	43	Anti-glaucoma surgery
**7**	3	Male	Right	ACG	1	-	-	3	31	Anti-glaucoma surgery
**7**	3	Male	Left	ACG	1	-	-	6	27	Anti-glaucoma surgery
**8**	2	Female	Right	Glaucoma suspect	7	35	Sulcus	50	28	Topical antiglaucoma medications
**8**	2	Female	Left	Glaucoma suspect	7	35	Sulcus	50	28	Topical antiglaucoma medications
**9**	2	Female	Left	Glaucoma suspect	4	24	In-the-bag	21	23	Topical antiglaucoma medications
**10**	2	Male	Right	Glaucoma suspect	2	38	In-the-bag	35	30	Topical antiglaucoma medications
**10**	2	Male	Left	Glaucoma suspect	2	38	In-the-bag	35	31	Topical antiglaucoma medications
**11**	2	Male	Right	Glaucoma suspect	6	31	Sulcus	41	31	Topical antiglaucoma medications
**11**	2	Male	Left	Glaucoma suspect	6	31	In-the-bag	41	32	Topical antiglaucoma medications
**12**	3	Female	Right	Glaucoma suspect	2	-	-	41	35	Topical antiglaucoma medications
**13**	2	Male	Right	Glaucoma suspect	3	35	In-the-bag	52	27	Topical antiglaucoma medications
**14**	2	Male	Right	Glaucoma suspect	2	43	In-the-bag	47	30	Topical antiglaucoma medications
**14**	2	Male	Left	Glaucoma suspect	2	43	Sulcus	47	31	Topical antiglaucoma medications

*IOL* Intraocular lens, *IOP max* Highest intraocular pressure measured, *OAG* Open-angle glaucoma, *ACG* Angle-closure glaucoma

The total incidence of postoperative steroid-induced ocular hypertension was10.4% (27 of 259 eyes). Steroid-induced ocular hypertension mainly occurred within 1 month after primary (16 eyes) and secondary (11 eyes) surgery. Two cases of open-angle glaucoma and six cases of glaucoma suspect eyes developed in patients with steroid-induced ocular hypertension after surgery.

### Risk factors for postoperative glaucoma-related adverse events

In Table 4, the results of the subsequent multivariate Cox proportional hazards analysis were used to determine the potential risk factors for postoperative glaucoma-related adverse events in groups 2 and 3. As no eyes in group 1 developed glaucoma-related adverse events, a risk factor analysis within this group was not possible.

**Table 4:** Univariate and multivariable Cox regression analysis of risk factors for glaucoma-related adverse events in the group 2 and 3

Potential risk factors	Univariate analysis		Multivariable analysis	
	***HR (95% CI)***	***P-value***	***HR (95% CI)***	***P-value***
**Sex**	0.803 (0.332–1.941)	0.626		
**Age at surgery**	0.979 (0.801–1.195)	0.833		
**FHCC**	4.461 (1.763–11.285)	0.002	50.463 (7.051–361.139)	< 0.001
**History of prematurity**	1.494 (0.493–4.521)	0.478		
**PFV**	1.489 (0.543–4.081)	0.439		
**Pre-op AL**	0.678 (0.479–0.959)	0.028	1.325 (0.750–2.341)	0.332
**Pre-op CCT**	1.013 (1.005–1.021)	0.002	1.021 (1.009–1.034)	0.001
**Pre-op HCD**	4.049 (2.105–7.752)	< 0.001	3.922 (1.558–9.804)	0.004
**Pre-op ACD**	0.126 (0.021–0.755)	0.023	0.256 (0.020–3.357)	0.300
**Pre-op LT**	3.559 (1.656–7.634)	0.001	3.745 (1.344–10.417)	0.012
**Secondary IOL implantation**	0.921 (0.321–2.641)	0.879		

*HR* Hazard ratio, *CI* Confidence intervals, *FHCC* Family history of congenital cataract, *PFV* Persistent fetal vasculature, *Pre-op* Preoperative, *AL* Axial length, *CCT* Central corneal thickness, *HCD* Horizontal corneal diameter, *ACD* Anterior chamber depth, *LT* Lens thickness, *IOL* Intraocular lens

In univariate analyses, family history of congenital cataract (HR, 4.461; 95% CI, 1.763–11.285; *P* = 0.002), preoperative AL (HR, 0.678; 95% CI, 0.479–0.959; *P* = 0.028), preoperative CCT (HR, 1.013; 95% CI, 1.005–1.021; *P* = 0.002), preoperative HCD (HR, 4.049; 95% CI, 2.105–7.752; *P* < 0.001), preoperative ACD (HR, 0.126; 95% CI, 0.021–0.755; *P* = 0.023), and preoperative LT (HR, 3.559; 95% CI, 1.656–7.634; *P* = 0.001) exhibited a significant association with the development of glaucoma-related adverse events. However, in multivariate analyses, only family history of congenital cataract (HR, 50.463; 95% CI, 7.051–361.139; *P* < 0.001), preoperative CCT (HR, 1.021; 95% CI, 1.009–1.034; *P* = 0.001), preoperative HCD (HR, 3.922; 95% CI, 1.558–9.804; *P* = 0.004), and preoperative LT (HR, 3.745; 95% CI, 1.344–10.417; *P* = 0.012) were significant risk factors; preoperative AL (*P* = 0.332) and preoperative ACD (*P* = 0.300) did not exhibit significant associations with the development of glaucoma-related adverse events.

Sex, age at surgery, history of prematurity, PFV, and secondary IOL implantation were not significantly correlated with postoperative glaucoma-related adverse events (all *P* > 0.05).

## Discussion

There seemed to be quite a lot of confusion on how to define glaucoma after congenital cataract surgery and the risk factors for glaucoma after congenital cataract surgery. The percentage of postoperative glaucoma-related adverse events varied according to the follow-up time [20] and the definition employed by the study [6]. The CGRN created a new classification system for childhood glaucoma that become the first International Consensus Classification. In this consensus, CGRN defines glaucoma after congenital cataract surgery [1]. IATS provided the definition of glaucoma suspect and glaucoma-related adverse events after congenital cataract surgery [19]. This study mainly focused on the occurrence of glaucoma after congenital cataract surgery. However, it was difficult for children to cooperate with visual field examination. In practical applications, the IATS standard was more in line with the actual situation in this study.

Some studies focused on ocular hypertension or glaucoma using long-term follow-up, whereas others have only assessed it within 1 year, resulting in vast differences in the results. A retrospective study conducted by Kirwan et al. that followed up patients for 23 years revealed that the rates of postoperative glaucoma were the highest within the first year after congenital cataract surgery [21]. Moreover, the incidence of glaucoma decreased dramatically 1 year after surgery. As the IATS included the initial data at the 1-year and 5-year follow-ups [15, 19], these studies have reported similar rates 10 years after surgery with increased rates of glaucoma-related adverse events [22]. The incidence of glaucoma, glaucoma suspect, and glaucoma-related adverse events after congenital cataract surgery observed in our study was lower than those reported in previous studies. One of the main reasons for the low incidence in our study may be that all procedures were completed by the same skilled senior specialist.

In our study, angle-closure glaucoma (3 eyes) occurred within 6 months, open-angle glaucoma (7 eyes) occurred within 2–6 years, and glaucoma suspect mainly occurred within 2–5 years after congenital cataract surgery. Based on the available evidence, the number of children diagnosed with glaucoma-related adverse events will likely increase as the duration of follow-up increases. In this context, age at surgery was not significantly correlated with postoperative glaucoma-related adverse events, which may be due to the low number of cases. However, the age at surgery in all cases in this study was less than 8 months.

Previous studies found that smaller corneal diameter, smaller ocular size, thicker CCT, and family history of congenital cataract were risk factors for postoperative glaucoma [4, 19, 23, 24]. Similarly, in this study, multivariate analysis showed that family history of congenital cataract, thicker preoperative CCT, and smaller preoperative HCD increased the risk for developing glaucoma-related adverse events. Most of the patients with glaucoma-related adverse events had family history of congenital cataract, but the number was too small. Therefore, the range of CI values was wider. Future studies will further expand the sample size. We found that thinner preoperative LT was also an important risk factor for glaucoma-related adverse events. Our previous study found that the thinner LT was an independent and valuable predictor of a preexisting posterior capsule defect in eyes with a congenital cataract [25]. In addition, we found significantly increased proinflammatory cytokine levels in the aqueous humour after congenital cataract extraction in children [26]. Thus, we hypothesised that leakage of crystalline lens materials due to a preexisting posterior capsule defect may be triggered to produce inflammatory cytokines. The outflow resistance of chronic trabeculitis increased due to lens remnants of paediatric cataracts or toxic substances from the vitreous or even chronic inflammation [21, 27]. These suggested a significant correlation between thinner preoperative LT and glaucoma-related adverse events. Future studies should explore the associations between glaucoma-related adverse events and preoperative measurements of LT and other risk factors.

Although the IATS did not define the clinical management of postoperative glaucoma, the 10-year IATS results reported that glaucoma surgery had been performed in 11 (44.0%) eyes with postoperative glaucoma, with seven eyes requiring a single operation at this time point [22]. Michaelides et al. reported that 7 (46.7%) of 15 glaucomatous eyes required surgical intervention for IOP control [28]. Kirwan et al. reported that all 7 pseudophakic eyes and 18 of 25 aphakic eyes with glaucoma underwent surgery for glaucoma control, with the Ahmed glaucoma valve being the first choice in all patients [21]. In our study, glaucoma-related adverse events were controlled with topical antiglaucoma medications in 18 eyes, while angle-closure glaucoma (3 eyes) needed anti-glaucoma surgery.

Glaucoma may be subcategorised by gonioscopy findings into open-angle or angle-closure glaucoma [1]. Most cases of postoperative glaucoma were of the late-onset open-angle type, accounting for 75.0%–93.8% of glaucoma cases after congenital cataract surgery [2, 29, 30]. In our study, all glaucoma suspect cases had open angles, with open-angle and angle-closure glaucoma accounting for 70.0% and 30.0%, respectively, of glaucoma cases after congenital cataract surgery. Open-angle glaucoma was diagnosed in seven eyes, all of which occurred within 2 to 6 years following surgery, whereas angle-closure glaucoma was diagnosed in three eyes, all occurred within 6 months after surgery. Angle-closure glaucoma with pupillary block after lensectomy was rare [29–31]. The children included in our study developed angle-closure glaucoma owing to peripheral anterior synechia rather than pupillary block because they had small corneas with shallow ACD and mydriasis twice a day after surgery. This indicated that pupillary dilation should be monitored carefully in eyes with small corneas.

The total incidence of postoperative steroid-induced ocular hypertension in our study was 10.4%. We found that the incidence of steroid-induced ocular hypertension, which progressed to glaucoma-related adverse events, was 29.6%. We suspect that sensitivity to steroid-induced ocular hypertension may be a high-risk factor for glaucoma-related adverse events.

The advantages of this study include its design divided into primary IOL implantation, secondary IOL implantation, and aphakia, as well as standardised definitions of glaucoma, glaucoma suspect, and glaucoma-related adverse events. In addition, we assessed the incidence of postoperative steroid-induced ocular hypertension and provided a better reference for postoperative medication. This study revealed the percentage of glaucoma-related adverse events at different time points after cataract surgery and could better detect the time of glaucoma onset after cataract surgery. However, our study was limited by its relatively short follow-up time.

In general, family history of congenital cataract, higher preoperative CCT, smaller preoperative HCD and thinner preoperative LT might increase the risk of postoperative glaucoma-related adverse events. As glaucoma-related adverse events following congenital cataract surgery were the leading causes of vision loss years after surgery, enhancing the understanding of the pathogenesis and potential risk factors is of paramount importance. We plan to collect longer-term follow-up data for all patients with congenital cataracts included in this study, which should further elucidate the mechanisms underlying glaucoma-related adverse events and may aid in preventing and managing cataracts in congenital patients.

## Conclusion

Family history of congenital cataract, thicker preoperative CCT, smaller preoperative HCD, and thinner preoperative LT are the main risk factors of postoperative glaucoma-related adverse events. Sensitivity to steroid-induced ocular hypertension may be a high-risk factor for glaucoma-related adverse events. Regular monitoring of children after cataract surgery with these risk factors may help ophthalmologists detect susceptible individuals and provide timely interventions in the clinic.

## Data Availability

The datasets generated during and/or analysed during the current study are available from the corresponding author on reasonable request.

## Abbreviations

IOP: Intraocular pressure
IOP max: Highest intraocular pressure measured
IOL: Intraocular lens
IATS: Infant Aphakia Treatment Study
CCT: Central corneal thickness
PFV: Persistent fetal vasculature
AL: Axial length; ACD: Anterior chamber depth
LT: Lens thickness
HCD: Horizontal corneal diameter
FHCC: Family history of congenital cataract
HR: Hazard ratio
CI: Confidence intervals
IQR: Interquartile range
Pre-op: Preoperative
CGRN: Childhood Glaucoma Research Network
OAG: Open-angle glaucoma
ACG: Angle-closure glaucoma
